# Surface modification of PEEK implants for craniofacial reconstruction and aesthetic augmentation—fiction or reality?

**DOI:** 10.3389/fsurg.2024.1351749

**Published:** 2024-02-28

**Authors:** Martin Kauke-Navarro, Leonard Knoedler, Samuel Knoedler, Can Deniz, Ali-Farid Safi

**Affiliations:** ^1^ Division of Plastic and Reconstructive Surgery, Department of Surgery, Yale New Haven Hospital, Yale School of Medicine, New Haven, CT, United States; ^2^ Craniologicum, Center for Craniomaxillofacial Surgery, Bern, Switzerland; ^3^ Medical Faculty, University of Bern, Bern, Switzerland

**Keywords:** PEEK, polyether ether ketone (PEEK), implant, face, craniofacial

## Abstract

Facial implantology, a crucial facet of plastic and reconstructive surgery, focuses on optimizing implant materials for facial augmentation and reconstruction. This manuscript explores the use of Polyetheretherketone (PEEK) implants in craniofacial surgery, highlighting the challenges and advancements in this field. While PEEK offers mechanical resilience, durability, and compatibility with imaging modalities, its biologically inert nature hinders integration with the host tissue, which may lead to complications. In this systematic review, our aim was to assess the current state of knowledge regarding the clinical evaluation of Polyetheretherketone (PEEK) implants in facial implantology, with a focus on craniofacial augmentation and reconstruction in human studies. Additionally, we explore and discuss surface and structural modifications that may enhance bioreactivity and reduce complications in PEEK implants. A systematic review identified 32 articles detailing the use of PEEK Patient-Specific Implants (PSIs) in 194 patients for both reconstructive and aesthetic purposes. Complications, including infections and implant failures, were reported in 18% of cases, suggesting the need for improved implant materials. The discussion delves into the limitations of PEEK, prompting the exploration of surface and structural modifications to enhance its bioreactivity. Strategies, such as hydroxyapatite coating, titanium coating, and porous structures show promise in improving osseointegration and reducing complications. However, the literature review did not reveal reports of coated or modified PEEK in facial reconstructive or aesthetic surgery. In conclusion, although PEEK implants have been successfully used in craniofacial reconstruction, their biological inertness poses challenges. Surface modifications, particularly hydroxyapatite coatings, provide opportunities to promote osseointegration. Future research should focus on prospective long-term studies, especially in craniofacial surgery, to investigate the stability of uncoated PEEK implants and the potential benefits of surface modifications in clinical applications. Patient-specific PEEK implants hold promise for achieving durable craniofacial reconstruction and augmentation.

## Introduction

Facial implantology is a scientific branch of plastic surgery that deals with outcome research and continued optimization of currently available implant materials for facial augmentation and reconstruction (1). Facial implants are part of a plastic surgeon's armamentarium to balance facial features, for example for aesthetic considerations and in patients with congenital or acquired mainly hard tissue asymmetries of the craniofacial skeleton. Aging-related predictable volume loss is a common reason for an acquired imbalance of facial features (2). Facial implants, often in combination with other orthognathic interventions, are frequently used to correct such facial asymmetries (3). Historically, autologous grafts were used, however, due to the unreliable nature of autologous grafts in terms of resorption (e.g., bone), donor site morbidity, prolonged operative times, limited availability, and difficult contouring, alloplastic materials have become standard of care for most elective augmentation cases in the adult patient. Alloplastic materials can be tailored to perfectly fit a patient's defect or augmentative needs via computer-assisted design and manufacturing processes to create patient-specific implants (PSI).

Various alloplastic materials, such as metals (e.g., titanium), polymers [silicone, porous polyethylene, polyether-ether ketone (PEEK)], and ceramics (e.g., Hydroxyapatite) can be used (4). However, all of these materials have associated disadvantages and the optimal facial implant has yet to be identified (4, 5). The ideal implant is chemically inert yet integrates with the host, can easily be contoured, is form-stable, and tolerates mechanical stress. In addition, resistance to infection or inflammatory reactions as well as the ability to osseointegrate are also important properties. PEEK implants have emerged as an excellent material for craniofacial augmentation and reconstruction. However, a major drawback of PEEK is its poor bioreactivity and bioinert nature. Thus, the material typically does not integrate with the host (i.e., poor osseointegration and low interfacial adhesion) causing a fibrous layer to form around the implant (6). The low bioreactivity of PEEK implants and the, therefore, often limited incorporation by the host have led to the development of modified PEEK implants. In the field of spinal surgery, the poor osseointegration and incorporation of the PEEK implant (cage) have resulted in fusion failures (7–10).

In this article, we investigate whether coated/modified PEEK implants have been scientifically evaluated for craniofacial augmentation and discuss material modifications that may further improve the clinical utility of this implant material.

## Methods

A comprehensive and systematic literature review was conducted by the authors of this review on PubMed/MEDLINE, GoogleScholar, CENTRAL, and Web of Science from database inception to 1st December 2023 for studies investigating whether coated/modified PEEK implants have been clinically evaluated for facial implantology. The following search terms were entered: (PEEK OR Polyetheretherketone) AND (FACE OR FACIAL OR ZYGOMA OR FRONTAL OR MALAR OR MAXILLA OR MANDIBLE OR TEMPORAL). The search format has been adjusted to the appropriate syntax of the respective database. Non-human studies or articles in a language other than English were excluded. Studies looking at isolated cranioplasties were excluded. Additionally, the currently available literature was reviewed to identify all relevant information on PEEK and related coating strategies to enhance the bio-acceptance of PEEK implants, specifically focusing on craniofacial application of the implant material. This study was conducted in accordance with the Preferred Reporting Items for Systematic Reviews and Meta-Analyses (PRISMA) 2020 guidelines (11). This study should be viewed as a descriptive review, as we did not perform a quantitative meta-analysis due to the heterogeneity in outcome parameters. Two reviewers (M.K.-N. and L.K.) independently screened the titles and abstracts of the articles using Covidence (12). A subsequent full-text review was performed manually for abstracts that had been considered eligible. Any disagreements were discussed with a third reviewer (A.-F.S.) and resolved by consensus.

## Results

We identified 32 articles reporting data on the use of PEEK PSIs for reconstruction or aesthetic augmentation of the facial skeleton in 194 patients (Table 1, Supplementary Figure S1) (13–23, 25–39, 41–44).

**Table 1:** Systematic review of the literature summarizing studies that report on PEEK implants used for reconstruction or augmentation of facial structures.

Author, year	Location	Indication	Implant design	Number of patients (mean age, range)*	Average follow up (range)	Fixation	Coating/PEEK adjustment/intraoperative adjustment	External manufacturer	Complications
Sainsbury et al. 2017 (13)	Malar	Aesthetic	PSI	3 (11, 7–16)	NA	Intraoral access, Screw and plate fixation,	–	NA	Globe compression requiring removal in 1 patient
Long et al. 2023 (14)	Mandibular	Reconstructive	PSI	1 (21)	14M	Screw and plate fixation, extraoral approach	–	NA	None reported
Thomas and Lee 2016 (15)	Mandibular—Chin Zygoma	Aesthetic	PSI	2 (NA)	NA	Transoral, subperiosteal. Interlocking system, screw fixated	Interlocking PEEK	Materialise	NA
Saponaro et al. 2023 (16)	Calvarium, Malar, Mandibular	Reconstructive/Aesthetic	PSI	28 (NA, 16–45)	NA	Intraoral/extraoral approaches, Screw and plate fixation	–	NA	None reported
Li et al. 2022 (17)	Mandible	Reconstructive	PSI	6 (60 ± 15, 45–75)	10–24M	Extraoral ± intraoral access, titanium screw fixation	–	State Key Laboratory for Manufacturing Systems Engineering, Xi'an Jiaotong University	Implant exposure 10 months after surgery requiring removal
Lv et al. 2022 (18)	Maxilla, Zygoma, Orbit	Reconstructive	PSI	12 (NA, 12–61)	2–20M	Extraoral + intraoral, screw fixation	–	Medprin	None reported
Anabtawi et al. 2021 (19)	Mandible (ramus, chin, body), zygoma	Reconstructive/Aesthetic	PSI	10 (NA, 17–54)	37 (11–61) M	Intraoral + transconjunctival (for orbito-zygomatic implant) Screw fixation	Interlocking PEEK	Synthes	30% complication rate (3 patients): edema, mental nerve paresthesia, sinusitis
Atef et al. 2022 (20)	Mandible (chin, angle)	Aesthetic	PSI	6 (NA, 22–34)	NA	Intraoral, screw fixation	–	*NA*	Chin implants w marked early edema; no other complications noted
Murnan et al. 2021 (21)	Malar, orbit, skull, mandible	Reconstructive/Aesthetic	PSI	32 (41, NA)	NA	Intraoral/transconjunctival	–	NA	Postoperative inflammation 28%, 9 patients required implant removal (infection/dehisence)
Nocini et al. 2022 (22)	Mandibular angle	Aesthetic	PSI	1 (25)	12M	Extra-oral approach, Titanium screw fixation	–	Synthes	None reported
Suresh et al. 2018 (23)	Frontal bone, Maxilla	Reconstructive	PSI	8 (45.75 ± 19)	10M	Screw fixation, no surgical detail on approach	–	NA	Infection in 1 case requiring explant
Jaervinen et al. 2019 (24)	Mandible, Zygoma, orbit	Reconstructive/Aesthetic	PSI	24 (31, range 16–72)	16 (2–63 months)	Screw fixation, Coronal, intraoral/extraoral, subciliary, transconjunctival	PEEK required intraoperative modification in 9 patients for perfect fit	Synthes	Wound dehiscence and infection in 2 cases, paresethesias in 6 cases, transient facial nerve (zygomatic branch) paralysis in 1 case
Olate et al. 2021 (25)	Mandibular angle	Aesthetic	PSI	21 (NA)	6M	Intraoral approach, single titanium screw	–	Arcomed	2 cases of implant infection treated with ABX and mouthwash
Shi et al. 2022 (26)	Zygoma	Reconstructive	PSI	1 (37)	12M	Extraoral approach	–	*Medprin Regenerative Medical Technologies Co*.	None reported
Doh et al. 2019 (27)	Temporal	Aesthetic	PSI	1 (53)	NA	Plates and screws	–	NA	None reported
Gerbino et al. 2015 (28)	Frontal, zygoma, oribit	Reconstructive	PSI	13 (52, 28–72)	18M	Screw/Plate fixation	PEEK required adjustment in 1 case	Synthes	Inadequate esthetic result in 2 cases
Khashaba and Shawky 2023 (29)	Temporal	Aesthetic	PSI	1 (40)	60M	Screw/Plate	–	NA	“Stretched skin” after 5 years, no immediate complication
Lee et al. 2023 (30)	Orbit	Reconstructive	PSI	1 (40)	12M	NA	–	NA	None reported
Bitner et al. 2021 (31)	Mandible, Zygoma	Reconstructive	PSI	1 (40)	12M	Hemicoronal, transparotid/cervical, Screw and plate fixation	–	Kelynium	None reported
Hussain et al. 2016 (32)	Zygoma, Orbit	Reconstructive	PSI	1 (67)	NA	Screw and plate fixation, Extraoral transfacial approach	–	NA	Hematoma postoperatively requiring revision
Guevera-Rojas et al. 2014 (33)	Zygomaticomaxillary complex	Aesthetic	PSI	1 (27)	2M	Extraloral approach, Screw fixation	–	Synthes	None reported
Coelho et al. 2022 (34)	Anterior Nasal spine	Aesthetic	PSI	1 (20)	NA	Extraoral, combined w rhinoplasty, screw and wire fixation	–	NA	None reported
Marbacher et al. 2011 (35)	Temporo-orbital	Reconstructive	PSI	1 (72)	60M	Plate and screw fixation, coronal approach	–	Synthes	None reported
Lavie et al. 2015 (36)	Zygoma	Reconstructive	PSI	1 (63)	15M	Plate and screw fixation, coronal approach	–	KLS Martin	None reported
Cung et al. 2022 (37)	Orbitomaxillary	Reconstructive	PSI	2 (35, 28–42)	3–8M	Plate and screw fixation, intraoral/transconjunctival	–	Synthes	Entropion, infraorbital nerve paresethesia
Camarini et al. 2011 (38)	Frontal	Reconstructive	PSI	1 (47)	18M	Plate and screw fixation, coronal approach	–	Synthes	None reported
Lai et al. 2010 (39)	Fronto-orbito-temporal defect	Reconstructive	PSI	1 (69)	7M	Coronal, miniplates and screws	–	Synthes	Revision POM 7, w skin breakdown and VP shunt dysfunction
Scolozzi et al. 2007 (40)	Orbito-fronto-temporal	Reconstructive	PSI	1 (42)	12M	Coronal, Screw and plate fixation	–	Synthes	Residual small temporal depression visible
Kim et al. 2009 (41)	Orbitomaxillary, frontal sinus	Reconstructive	PSI	4 (19, 11–29)	14–20M	NA	–	Synthes	None reported
Jalbert et al. 2014 (42)	Frontal bone, orbit	Reconstructive	PSI	5 (50, 30–69)	3–12M	Intraoral, extraoral, Sccrew and plate fixation,	–	Synthes	Seroma, w/o need for intervention, diplopia that resolved in 2 cases
Scolozzi 2012 (43)	Orbit, mandible	Reconstructive	PSI	2 (29)	12–24M	Transconjunctival, intraoral, plates and screws	–	NA	None reported
Berrone et al. 2015 (44)	Mandible	Aesthetic	PSI	1 (27)	8M	Extraoral, screw fixation	–	Synthes	None reported

* if applicable.

None of the articles described the use of coated PEEK implants or discussed surface modifications. The use of interlocking PEEK was documented in 2 cases. Intraoperative modifications were reported in 2 articles.

The earliest article was published in 2007 by Scolozzi et al. reporting on the use of PEEK PSIs for the reconstruction of an orbito-fronto-temporal post-traumatic defect (40). The majority of articles (18/32; 56%) described the use of PEEK PSIs for reconstructive purposes (e.g., following tumor resection or for the reconstruction of posttraumatic defects), while 10 articles (31%) described primarily aesthetic indications (e.g., congenital deficiencies). Reconstruction/augmentation of the zygoma was described in 14 cases, mandible in 13 cases, orbit in 9 cases, frontal bone in 7, temporal bone in 3, and anterior nasal spine in 1.

In 4 articles, both reconstructive and aesthetic applications were described. The majority of articles were case reports (19/32; 60%). The age range was 7–75 years. Where available, follow-up time was recorded and found to be heterogeneous, ranging from 2 months to 5 years, with a calculated median of 12 months. Various complications in 36 patients (36/194; 18%) were reported, including globe compression requiring removal (1 patient), nerve paresthesia (10), infections/exposure requiring removal (12), facial nerve compression (1), dehiscence/infection not requiring removal (4), inadequate aesthetic result (3), hematoma/seroma (2), entropion (1), and diplopia not requiring removal (2). Details on the manufacturer were available in 20 cases, most of which used Synthes as their PEEK manufacturer (12).

## Discussion

### Polyetheretherketone (PEEK)

PEEK, a non-resorbable thermoplastic polymer, has become a well-known alloplastic material for cranial vault reconstruction and facial augmentation. PEEK has high mechanical resilience (elastic modulus comparable to cortical bone), is durable and retains its shape, features low thermal reactivity and bioreactivity, and is compatible with standard imaging modalities (i.e., MRI and CT). Additionally, PEEK implants allow intraoperative adjustments by any standard high-speed burrs and patient-individualized plate/screw fixation is easily possible.

However, due to its chemical composition, PEEK implants are biologically inert (7, 45). The high chemical stability is an advantage due to low toxicity, low thermal reactivity, and low peri-implant inflammatory reactions (45). A major drawback of PEEK is its poor bioreactivity and bioinert nature as well as its low antibacterial properties. Accordingly, the material typically fails to integrate with the host (poor osseointegration and low interfacial adhesion), leading to the formation of a fibrous layer around the implant (6). To date, long-term and large-scale studies specifically in the field of facial implantology are absent (Table 1). However, experience in other fields of orthopedic surgery may help guide implant design and selection in the field of facial implantology.

**Figure 1:**
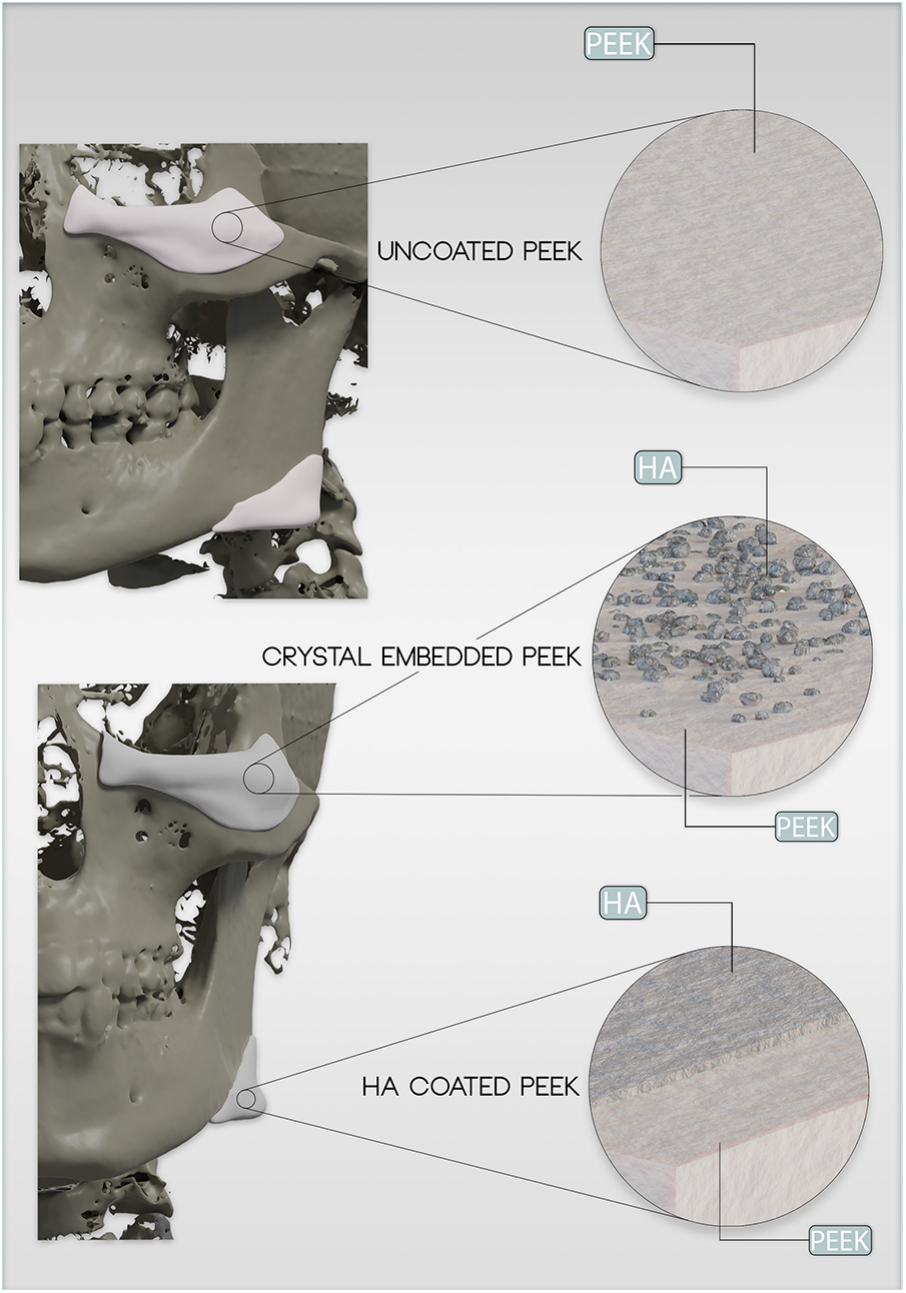
Example of zygomna and mandibular angle implant augmentation using patient-specific PEEK implants. Schematic demonstration of different PEEK-augmentation strategies using HaP embedding or surface coating. Embedded PEEK was designed to limit the possibility of damaging the HaP surface coat during the process of implant insertion.

The low bioreactivity of PEEK implants and, hence, frequently limited incorporation by the host have prompted the development of modified PEEK implants (Figure 1). In the field of spinal surgery, the poor osseointegration and incorporation of the PEEK implant (cage) have caused fusion failures (7–10). Further studies in the area of craniofacial surgery revealed a total complication rate of nearly 16% with 9% implant failure in cranioplasties with PEEK (46). In another study, the infection rate was reported to be as high as 8% (24). PEEK implants may also favor biofilm formation and, thus, bacterial adhesion compared to alternative materials (47).

To improve the characteristics of PEEK implants and the bioreactivity, integration, and ability of the host to control PEEK infections, a series of strategies have been employed which will be reviewed below (8, 48). Surface and structural modifications of existing implants have been described to improve osteoinductive and osteoconductive properties. For example, in spinal surgery, Ti-coated PEEK cages have been shown to offer improved osseointegration compared to uncoated PEEK (49). Our systematic review of the literature did not identify any reports of coated or modified PEEK application in facial reconstructive or aesthetic surgery. However to improve the characteristics of PEEK implants used for craniofacial augmentation and reconstruction, such strategies may need to be applied in order to further improve patient outcomes. Below we discuss some common straties used for ex situ PEEK modification.

## Surface and structural modifications

### Bioceramic coatings

HaP is a bioceramic material that consists of calcium and phosphate and presents the natural inorganic component of human bone (50). HaP based implants are known to promote superior cellular attachment and integration (osteoconduction, osseointegration) compared to other alloplastic implants (51). Thus, HaP-derived implants are more likely to be incorporated by the recipient when compared to for example titanium or silicone implants. In a study using HaP for facial augmentation, implant volume stability of 99.7% was shown at 2 year follow up (52).

Hence, HaP is considered an excellent material for surface coating (or embedding) of PEEK implants (Figure 1). Hydroxyapatite (HaP) coating or embedding has been studied to improve the bioactivity of PEEK implants. In general, HaP is the most commonly used surface modification for PEEK implants used for spinal surgery (8).

*In vivo* experiments have demonstrated improved osseointegration of HaP-coated PEEK implants (53). In an *in vivo* pre-clinical model, HaP-coated PEEK implants were reported to regenerate a larger bone volume on the implant surface and provide superior bone-to-implant contact (53).

Johannson et al. demonstrated that spin-coated PEEK with HaP implanted into rabbit femur/tibia was able to increase removal torque and improve contact between PEEK and native bone (54). In another study, Hahn et al. documented that aerosol-deposited HaP on PEEK enhanced bioreactivity both *in vivo* and *in vitro* (55). *In vivo* studies using a rabbit tibia defect model indicated that HaP coating improved the bone-to-implant contact interface (55). Clinically, HaP-coated PEEK cages were used for anterior cervical spine fusion and demonstrated higher fusion rates compared to uncoated PEEK implants (56).

Other materials that were proposed for PEEK coating are HaP/SiO_2_ (silicon dioxide) which showed improved osseointegration (57). Other proposed bioceramic coatings include zirconium-dioxide (ZrO_2_), which is widely used in dental implantology, offering excellent biocompatibility. It, therefore, may also represent a promising option for coating PEEK implants (58). In this context it is worth mentioning that ZrO_2_-coated dental implants were found to improve the bone-to-implant contact ratio when compared to uncoated implants (59). In addition, ZrO_2_ exhibits antibacterial properties and promotes osseointegration (8).

### Titanium-coated PEEK

Titanium implants are commonly used in craniofacial surgery, for example for orbital reconstruction. Most plating systems are made from titanium due to its high biocompatibility, excellent compatibility with imaging modalities, mechanical strength, and corrosion resistance.

In pre-clinical studies, *in vitro* co-culturing of 3D-printed Ti-coated PEEK implants with pre-osteoblasts led to improved attachment, proliferation, and differentiation on Ti-coated PEEK samples (60). In an *in vivo* model, these findings were confirmed, with evidence of improved biocompatibility and bone regeneration on Ti-coated PEEK implants compared to uncoated PEEK implants (60).

In the setting of spine surgery, titanium-coated PEEK cages have shown improved bony fusion rates compared to standard PEEK cages, suggesting improved stability and integration of the implant (56, 61).

### Structural modification compared to smooth surface PEEK

Limited osseointegration may be attributed to the fact that PEEK implants are typically smooth surface implants (6). Porous PEEK with 300–400 um porous features (created by melt extrusion technique) was developed with the intent of facilitating bony ingrowth similar to porous polyethylene implants (62). *In vivo* pre-clinical experiments in a rat model revealed bony ingrowth into the implant with improved osseointegration compared to smooth surface PEEKs. Notably, porous PEEK retained the majority of the solid version's mechanical strength (about 74% of the fatigue resistance of smooth, non-porous, PEEK implants).

An interesting variation are 3D-printed PEEK-hydroxyapatite microporous prints (average pore size of 280 um) which offer improved *in vitro* cellular attachment and proliferation compared to standard PEEK, although a loss of tensile strength with an increase in HaP component and porosity was seen (48, 63).

Torstrick et al. investigated the effect of titanium-coated PEEK (roughened titanium surface) and porous PEEK implants in comparison to smooth PEEK: porous PEEK implants exhibited the best osteoinductive and osseointegrative properties when compared to smooth and titanium-coated PEEK implants (6).

Nanostructured PEEK implants are a novel biotechnological advancement with the aim of further promoting PEEK implant bioreactivity and osseointegration (8). Nanostructures involve specific topographic modifications, including the creation of pores and pillars on the surface of PEEK implants. Zheng et al. reported that fine surface structure modification can influence cellular behavior and may help promote osseointegration of modified PEEK implants (8).

## Conclusion

PEEK implants have successfully been used for craniofacial reconstruction. However, reporting on the outcome is highly heterogeneous with short follow-up time. Additionally, we saw a relatively high complication rate (18%) which may be reflective of the high number of complex case reports that focus on challenging cases. Experience with PEEK implants in other specialties suggests that the biologically inert nature of PEEK implants and the resulting poor integration may have significant downsides. Long-term studies on PEEK implants in craniofacial surgery are warranted to investigate the stability of uncoated PEEK implants.

In pre-clinical studies testing surface modifications, hydroxyapatite has been demonstrated to improve the integration of PEEK implants. Future research is essential to analyze the benefit of PEEK surface coating for clinical applicability—especially in craniofacial surgery. Patient-specific PEEK implants represent a unique opportunity to achieve long-lasting desired reconstruction and augmentation of the craniofacial skeleton.

## Supplementary material

Supplementary Figure S1 Prisma flow diagram of the herein presented systematic review.
