# Supplementary figures and images for: Surface modification of PEEK implants for craniofacial reconstruction and aesthetic augmentation—fiction or reality?
Source: Front Surg. 2024 Feb 28;11:1351749. doi: 10.3389/fsurg.2024.1351749 (PMC10936457; doi:10.3389/fsurg.2024.1351749)

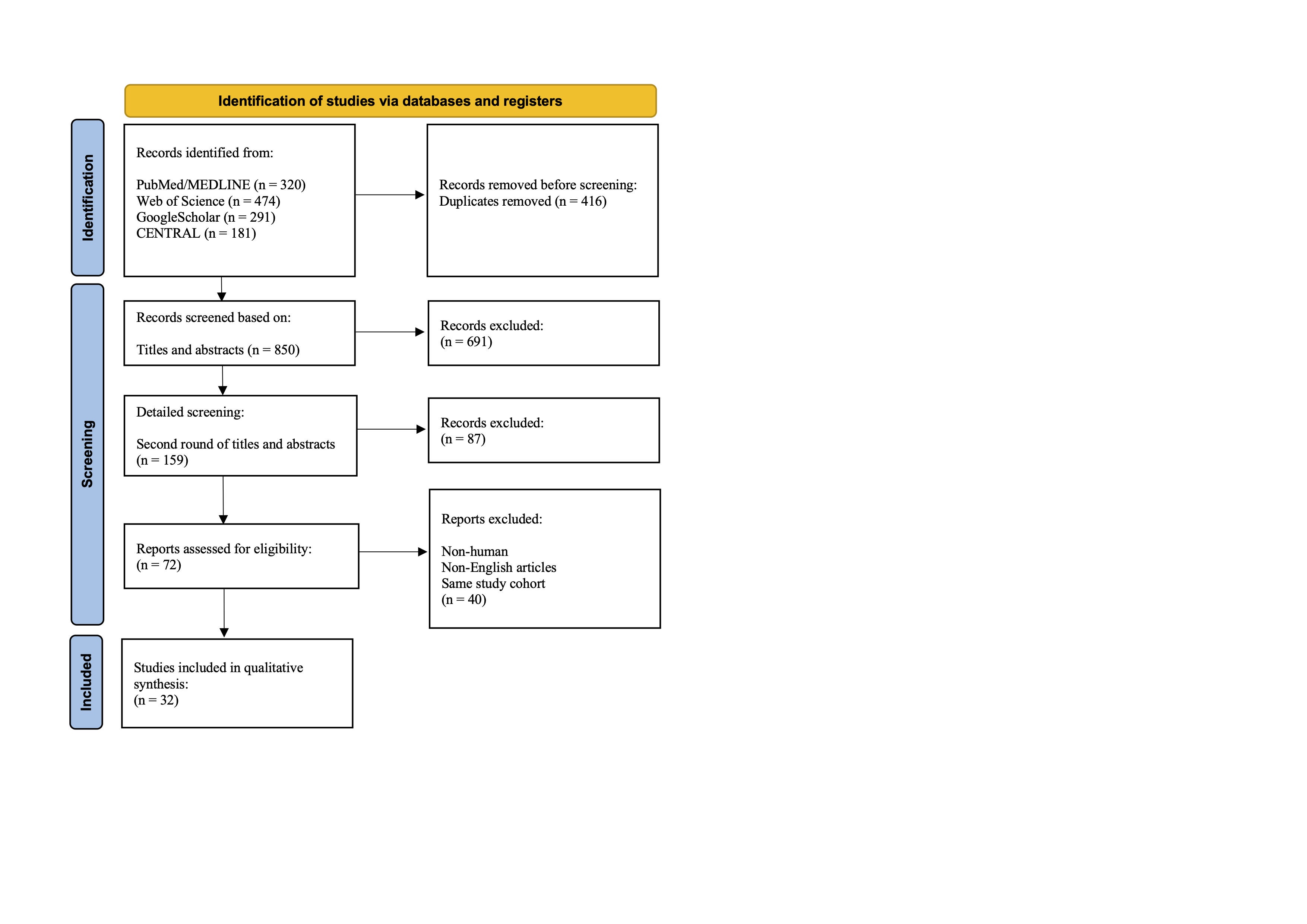

Supplement: Supplementary Figure S1 — Prisma flow diagram of the herein presented systematic review. [file Image1.jpg]
